# A Comparative Analysis of Symmetry Indices for Spatiotemporal Gait Features in Early Parkinson’s Disease

**DOI:** 10.3390/neurolint15030070

**Published:** 2023-09-07

**Authors:** Erasmia Giannakou, Styliani Fotiadou, Vassilios Gourgoulis, Georgios Mavrommatis, Nikolaos Aggelousis

**Affiliations:** 1 Department of Physical Education and Sport Science, School of Physical Education and Sport Science, Democritus University of Thrace, 69100 Komotini, Greece; egiannak@phyed.duth.gr (E.G.); sfotiado@phyed.duth.gr (S.F.); vgoyrgoy@phyed.duth.gr (V.G.); 2 Neurology Department, Democritus University of Thrace, 68100 Alexandroupolis, Greece; 3 Department of Physical Education and Sport Science, School of Physical Education and Sport Science, Aristotle University of Thessaloniki, 57001 Thessaloniki, Greece; gmavromma@phed.auth.gr

**Keywords:** gait analysis, asymmetry, Parkinson’s disease

## Abstract

This study compared the five most commonly used equations for calculating gait symmetry in discrete variables among Parkinson’s disease patients. Twelve patients (five women and seven men) performed ten consecutive gait trials on a 10 m walkway. Gait data were collected using eight optoelectronic cameras (100 fr/s). The analysis focused on various spatiotemporal parameters, including cadence, step time, stride time, single support, double support, walking speed, step length, stride length, step width, and foot angle. Five symmetry indices were calculated for each trial rather than averaging the ten recorded trials. The variability in and reliability of each symmetry equation were assessed using the coefficient of variation (CV) and intraclass correlation coefficient (ICC), respectively. Additionally, Bland–Altman plots were produced to visualize the agreement between each pair of methods for each spatiotemporal parameter. The results revealed that the symmetry ratio method exhibited lower variability and higher reliability compared with the other four indices across all spatiotemporal gait parameters. However, it was found that the reliability of a single trial was generally poor, regardless of the symmetry calculation formula used. Therefore, we recommend basing measurements of gait asymmetry in Parkinson’s disease on multiple trials.

## 1. Introduction

Parkinson’s disease is a progressive, degenerative disorder of the central nervous system that significantly affects the movement of patients. Cardinal motor symptoms include tremor, rigidity, and bradykinesia (i.e., slowness of movement), which modify the balance and gait of Parkinson’s patients. Especially, gait deficits that mainly depend on motor programming, such as symmetry, are evident from the early onset of the disease [1].

In the context of walking patterns, symmetry refers to the consistent movement exhibited by either the lower or upper limbs over the course of a walking cycle [2,3,4]. While it is often simplified that a normal and physically capable gait is symmetrical, the reality is that natural asymmetry exists, particularly in the lower limbs, attributable to the distinct roles each limb plays during walking [3]. This inherent asymmetry acts as the benchmark for distinguishing between regular and abnormal walking, based on notable deviations.

In Parkinson’s disease, increased gait asymmetry is observed in temporal characteristics even in the early stages of the disease [1]. This evidence suggests that gait asymmetry may be present well before the patient seeks medical attention. Early recognition of such symptoms may help to diagnose the condition at an earlier stage. Nowadays, technology has significantly progressed to detect subtle changes in gait characteristics, like symmetry in spatiotemporal parameters, using low-cost and wearable tools. In addition to early detection, such applications could help monitor rehabilitation, disease progression, and even medication management.

Although this asymmetry may not be associated with the level of asymmetry observed in motor symptoms, such as tremor or rigidity, it tends to worsen as the disease progresses and is clearly influenced by the cognitive loading [5]. Dual-tasking serves as a clinical marker for cognitive load and has been shown to affect upper limb symmetry during the gait of people with Parkinson’s disease [6]. The effect on the lower limbs is suggestive and depends on the parameter examined [7,8,9].

Therefore, assessing gait symmetry in individuals with Parkinson’s disease constitutes an important factor not only for the evaluation and diagnosis of prodromal motor symptoms but also for the cognitive control that is required for walking. Furthermore, impaired lower limb symmetry is associated with balance problems, falls, and freezing episodes in people with Parkinson’s disease. Consequently, evaluating gait symmetry becomes critical for the assessment of individuals with neurological impairments [10].

According to Patterson et al. [11], symmetry measures have two components: (a) the equation chosen to calculate symmetry and (b) the spatiotemporal gait parameter being assessed.

Concerning the symmetry equation, different indices have been suggested by different research groups in the last decades [12,13,14,15,16]. Despite the plethora of proposed methods, all of them have weaknesses and limitations, especially when applied to different types of variables [17].

The lack of a universally accepted approach for calculating gait symmetry has led to difficulties when comparing studies due to variations in the formulas used and the range of variables assessed. Particularly in clinical practice, there is a need for a fast, easy, precise, and reliable tool that can be utilized to evaluate notable asymmetries in the lower extremities using simple discrete parameters.

Over the last fifteen years, several studies compared multiple indices and variables to identify the most appropriate symmetry equation for each gait parameter. Most of these studies focused on healthy adults [14,18,19] or children [17], with only a limited number investigating injured individuals [17] or patients with neurological diseases [11,20]. More recently, Hill and Nantel [21] used a different perspective and conducted a preliminary evaluation of the sensitivity of discrete symmetry metrics. They used power simulation techniques and validated their results by introducing mechanically induced asymmetries in data collected from healthy individuals.

Focusing on neurological conditions, Sant’Anna et al. [20] introduced a novel continuous method for calculating gait symmetry in Parkinson’s disease patients. Their objective was to differentiate between early-to-mild Parkinson’s disease patients and healthy elderly individuals. To achieve this, they compared a new proposed index with six commonly used symmetry calculation methods (three discrete and three continuous) using gyroscope data from the upper and lower limbs. They concluded that the proposed continuous method was suitable for the particular group of patients and dataset examined.

Another study by Patterson et al. [11] investigated whether different expressions of symmetry for discrete data yield distinct results in spatiotemporal gait data from individuals post-stroke. They selected four commonly used symmetry calculation equations (symmetry ratio, symmetry index, gait asymmetry, and symmetry angle) and applied them to five typical spatiotemporal parameters. Their analysis revealed no significant differences among the examined equations. The authors concluded that the choice of gait parameter used in the equation was the most crucial factor in assessing post-stroke gait symmetry. They recommended the use of the symmetry ratio because of its simplicity and suggested using step length, swing time, and stance time as the parameters for inclusion in the equation.

Since uncertainty exists as to the best index of gait asymmetry in Parkinson’s disease, the present study aimed to compare the most commonly used equations for quantifying gait symmetry in terms of variability, reliability, and agreement, focusing on gait spatiotemporal parameters in Parkinson’s disease patients.

The primary novelty of this research lies in its departure from prior studies, as it embraces a comprehensive strategy by investigating reliability, variability, and agreement concurrently within a single inquiry. Additionally, our study covers a wide range of spatial and temporal factors concerning walking patterns, aligning with the significance of these specific factors highlighted by Patterson [11]. Lastly, as far as we know, this represents the first attempt to compare symmetry indices in the walking patterns of individuals in the early stages of Parkinson’s disease. This holds notable importance as these differences are nuanced during this initial phase, and quantifying the degree of symmetry could provide valuable understanding for diagnosing the disease.

## 2. Materials and Methods

### 2.1. Subjects

Twelve patients (seven men) with a medical diagnosis of PD enrolled in this study (age: 66.25 ± 7.9 years, height: 165.2 ± 13.4 cm, and time since diagnosis: 2.97 ± 3.19 years, UPDRS: 19.58 ± 14.28). Disease severity was quantified using the Unified Parkinson’s Disease Rating Scale (UPDRS) by an experienced neurologist. Individuals who met the following exclusion criteria were not included in the study: (a) reliance on assistance for ambulation, (b) presence of other known neurological disorders, (c) history of head trauma, and (d) neurological, muscular, and skeletal injuries or deformities that could affect gait function. Prior to data collection, each participant was informed about the experimental protocol and this study’s objectives, and they provided written consent.

With the exception of one subject, all others were receiving anti-Parkinsonian medication. This study’s main objective was to compare symmetry assessment methods rather than measure the symmetry extent of Parkinson’s patients. Symmetry indices were computed using consistent trials across all analyzed patients, rendering potential medication or time effects on limb symmetry indices insignificant. Nevertheless, data collection was conducted when participants were in the “on” state, approximately 1–1.5 h after their most recent medication dose.

### 2.2. Procedure

The participants arrived in the laboratory individually, without having previously engaged in any intense physical activity. Upon arrival, anthropometric data were recorded, and a researcher placed sixteen reflective markers on their lower limbs, according to the PlugInGait protocol. The same researcher performed the marker placement for all participating patients.

To familiarize themselves with the equipment and measurement procedure, the subjects performed 10 gait trials at their natural speed. A timing device with photocells recorded walking speed during these trials, and the average speed for each individual was calculated using these data. A moving stick was then adjusted to move along a walkway at the calculated average speed for each participant.

During the measurement phase, the participants performed 10 consecutive valid gait trials on a 10 m walkway at the predetermined speed set by the aforementioned moving stick. This ensured that all trials were performed at the same natural speed for each subject.

### 2.3. Gait Analysis

Six optoelectronic cameras (Vicon MX3, Vicon Motion Systems, Oxford, UK) with a resolution of 0.3 megapixels and operating at a frame rate of 100 fr/s were used to record the movement of the participants’ lower limbs during gait. In addition, two Kistler forceplates (models: 9281B11 and 9281CA, Kistler Instruments, Winterthur, Switzerland), measuring 60 × 40 cm were positioned in the middle of the walkway. These forceplates had a sampling rate of 1000 Hz and were used to detect key events within each gait cycle, including foot strike and foot-off instances. The laboratory setup was arranged so that the forceplates were positioned at the center of the camera cycle.

For each of the ten recorded trials, the following spatiotemporal parameters were calculated: cadence in steps per minute, stride time in seconds, step time in seconds, single support time in seconds, double support time in seconds, stride length in centimeters, step length in centimeters, step width in centimeters, foot angle in degrees, and walking speed in meters per second.

### 2.4. Data and Statistical Analysis

Five different indices were used (see Table 1 for equations) to estimate gait symmetry: symmetry index (SI), gait asymmetry (GA), symmetry ratio (SR), ratio index (RI), and symmetry angle (SA) (see Table 1 for reference). In most of these equations, a value of 0% indicates full symmetry, while values exceeding 100% indicate full asymmetry. However, the SR deviates from this pattern, as a value of 1.0 signifies full symmetry, and there is no upper limit for the value indicating full asymmetry.

The calculations for all five symmetry equations were derived from identical patient data, thereby maintaining consistent measurement accuracy across the entire set of equations. Additionally, all patients underwent measurements utilizing the same equipment and within identical conditions. The cameras within the laboratory were affixed to the ceiling and remained in a static position with unchanged settings, such as focus, sensitivity, and resolution, throughout the measurement process. Furthermore, the laboratory was situated underground with predominantly artificial lighting to ensure consistent illumination.

Unlike other conditions like stroke or injury that involve asymmetric gait, we did not differentiate between the affected and non-affected lower limbs in Parkinson’s disease patients. This decision was based on the variability observed in the UPDRS scale [22] where not all Parkinson’s patients displayed a clear distinction between the right and left sides. The calculation of symmetry using the different equations was performed on a trial-by-trial basis rather than averaging the ten recorded trials. This was due to the prevalent formulation of symmetry equations in this study as fractions. Our strategy for handling fractional equations hinged on how we intended to address the diversity and discrepancies in our data. Using trial averaging serves as a means for mitigating deviations and extreme values from individual trials, thereby mitigating the impact of random errors and fluctuations. On the contrary, using a trial-specific calculation for fractional equations treats each trial as an independent occurrence. This approach effectively accentuates the diversity and spread of data across trials. Given that our research aimed to evaluate the variability in data derived from various fractional equations, we opted to adhere to the latter approach.

To assess the variability in each symmetry equation when estimating gait symmetry for each spatiotemporal parameter, the coefficient of variation (CV) was used. To evaluate the reliability of each symmetry index, we additionally computed intra-class correlation coefficients (*ICC*) between the 10 different gait trials (*ICC*_10_) and for a single trial (*ICC*_1_) using a two-way ANOVA model according to the below Equations (1) and (2) [23]:

(1) $$ICC=\frac{MS_{s}-MS_{i}}{MS_{s}}$$

(2) $$ICC_{1}=\frac{MS_{s}-MS_{i}}{MS_{s}+(N/K-1)\cdot MS_{i}}$$

where *MS_s_* is the mean square between trials, *MS_i_* is the mean square of the interaction, *N* is the number of trials, and *K* is the number of desirable trials for which the coefficient of reliability is estimated.

To evaluate the agreement among the various symmetry equations, we normalized each symmetry value by dividing it by its peak observed value for the specific symmetry equation and spatiotemporal gait parameter. We then used Bland and Altman’s approach [24] to assess the agreement for all possible pairs. Prior to conducting the analysis, we subtracted the absolute symmetry ratio values (calculated with the higher value as the numerator) from 1 to address the issue of reverse grading compared to the other equations.

## 3. Results

Table 2 presents the mean and SD values for all symmetry indices calculated (SI, GA, SA, SR, RI), as well as for each spatiotemporal parameter.

The symmetry indices derived from the different equations displayed similar magnitudes (within their respective reference ranges) for each spatiotemporal parameter. Moreover, these indices demonstrated comparable levels of gait symmetry across most spatiotemporal parameters, except for foot angle. Notably, the foot angle exhibited poorer symmetry in relation to all other spatiotemporal parameters.

**Figure 1:**
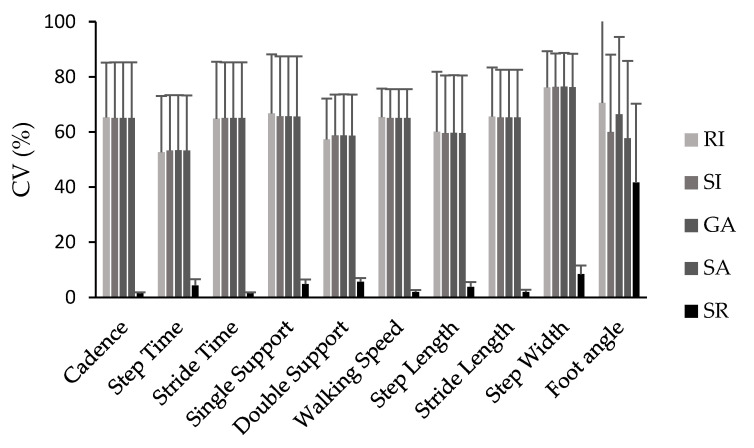
Coefficient of variation (CV%) values for all symmetry indices (SI, GA, SA, SR, RI) and spatiotemporal parameters.

Likewise, the variability in symmetry values was comparable among the symmetry index (SI), gait asymmetry (GA), ratio index (RI), and symmetry angle (SA) for most spatiotemporal parameters except for foot angle, which presented the highest variability. Although the symmetry angle index generated lower symmetry values compared with SI, GA, and RI, it demonstrated similar variability values. In general, the variability in most symmetry indices was large except for the symmetry ratio (SR), which consistently exhibited the lowest and only accepted range of variation across all symmetry indices and parameters assessed (Figure 1).

**Figure 2:**
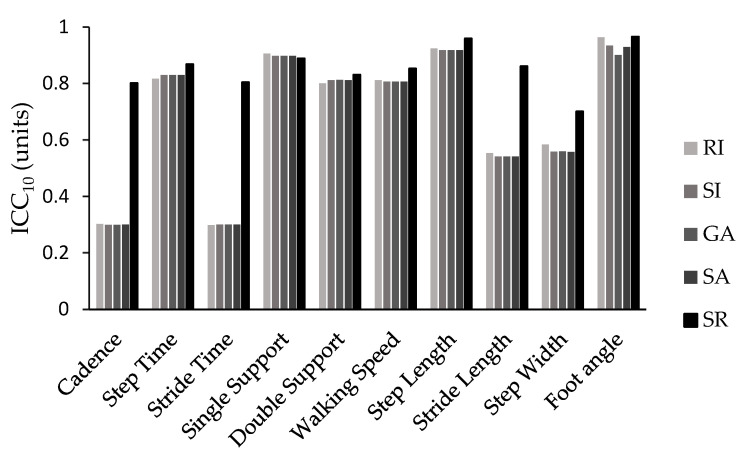
*ICC*s values for all 10 trials (*ICC*_10_) for all symmetry indices (SI, GA, SA, SR, RI) and spatiotemporal parameters.

**Figure 3:**
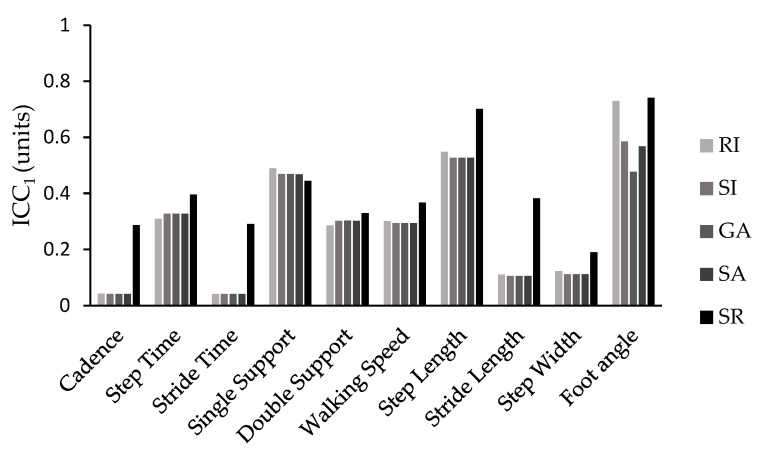
*ICC*s values for a single trial (*ICC*_1_) for all symmetry indices (SI, GA, SA, SR, RI) and spatiotemporal parameters.

The *ICC* values followed a similar pattern, with the SI, RI, GA, and SA showing similar values, while the SR exhibited the best values. This pattern was particularly evident when considering *ICC* values for the majority of the spatiotemporal parameters. The only exceptions were single-support time and double-support time, where all the indices yielded similar *ICC* values, and walking speed and foot angle, where the RI and SR demonstrated comparable and higher *ICC* values compared with the other methods used in this study (Figure 2 and Figure 3).

The intraclass correlation coefficient calculated for the sum of the ten trials (*ICC*_10_) showed excellent reliability (greater than 0.80) for step time (0.817–0.868), single support (0.889–0.906), double support (0.800–0.831), walking speed (0.806–0.853), step length (0.918–0.959), and foot angle (0.901–0.966) across all symmetry calculation methods used. Additionally, *ICC*_10_ showed excellent reliability for cadence (0.801), stride time (0.868), and stride length (0.861) and good reliability for step width (0.701) when symmetry was calculated using the symmetry ratio equation. In contrast, the use of any other formula resulted in moderate reliability for stride length (0.541–0.553) and step width (0.557–0.584) and poor reliability for cadence (0.299–0.302) and stride time (0.298–0.300).

The Bland–Altman plots demonstrated that the differences between the indices for all tested parameters mostly fell within the 95% confidence intervals (Figures S1–S3, Supplementary Material). Notably, a similar pattern was observed in the Bland–Altman plots for the SI-RI, SR-RI, SA-RI, and GA-RI pairs across all assessed spatiotemporal parameters except for step time, where the same pattern only appeared in the SI-RI, SA-RI, and GA-RI pairs. Similar plot patterns were also observed in the SR-SA and SR-GA pairs (excluding stride length and foot angle), as well as the SI-GA and SA-GA pairs (for cadence, step time, stride time, double support, and foot angle). No difference was detected between SI and SA for cadence, step time, or stride time parameters.

Table 3 and Table 4 display mean values and 95% limits of agreement (lower and upper limits) calculated using a Bland–Altman analysis for each possible equation pair and all evaluated spatiotemporal parameters. The bias was generally small for all evaluated equation pairs and all the considered parameters. Similar values were observed between certain symmetry equation pairs, consistent with the patterns observed in the Bland–Altman plots. The analysis revealed similar values between the SR-SA and SR-GA pairs for all parameters except foot angle. Likewise, the SI-RI, SA-RI, and GA-RI pairs showed similar values for single support, double support, stride length, step length, and step width and even exactly the same values for cadence, stride time, and walking speed. Furthermore, the SI-GA and SA-GA pairs had exactly the same values for cadence and stride time.

## 4. Discussion

Parkinson’s disease patients experience a range of motor symptoms, most of which initially manifest unilaterally, especially in the early stages of the disease. The asymmetrical appearance of symptoms in different body parts may serve as a significant diagnostic criterion for this neurological degenerative disorder. Therefore, the early, consistent, and reliable assessment of symmetry, especially during common activities such as walking, is of utmost importance. Currently, there are no universally accepted indices for gait symmetry. Therefore, this study examined the suitability of five commonly used equations for the evaluation of gait symmetry in Parkinson’s disease patients, focusing on discrete gait variables. We hypothesized that (a) all the symmetry methods used would show similar reliability and variability, irrespective of the spatiotemporal parameter being measured, and (b) there would be a high level of agreement between the symmetry indices when tested in pairs for each parameter.

Three of the compared indices, namely, the symmetry index (SI), gait asymmetry (GA), and ratio index (RI), yielded similar symmetry values for all examined parameters except for foot angle. Notably, one of the study participants exhibited noticeable asymmetry in the foot angle of their lower limbs. The ratio index produced significantly higher maximum value for foot angle than the other two calculation methods. Since these methods are not bounded and can exceed 100%, it appears that the ratio index expresses asymmetry differently due to its different rating scale.

Symmetry angle produced lower symmetry values compared with SI, GA, and RI. However, they all exhibited a similar range of coefficient of variation (CV) values across all assessed spatiotemporal parameters. The symmetry ratio (SR) also showed lower values of symmetry, likely due to its distinct grading system (full symmetry = 1), compared with all the other methods (full symmetry = 0%).

In terms of variability, the only equation that produced low and acceptable variability values (<10%) for most parameters, was the symmetry ratio. In general, the variability in the symmetry values was large (ranging from 8.5% to 120%) and comparable for the remaining calculation methods (SI, GA, SA, and RI) across all spatiotemporal gait parameters. The parameters with the least variability, when symmetry was calculated using the SR method, were cadence, stride time, walking speed, and stride length.

Similarly, the intraclass correlation coefficient (*ICC*) followed a similar pattern for the SI, GA, SA, and RI with few minor exceptions. The SR consistently presented higher *ICC* values for almost all spatiotemporal parameters compared with the rest of the symmetry equations. It was also the only expression that consistently demonstrated good (*ICC*_10_ = 0.60–0.80) or excellent (*ICC*_10_ > 0.80) reliability when *ICC* was calculated from the average of the ten trials across all gait parameters. When reliability was assessed for a single trial (*ICC*_1_), most variables showed poor (*ICC*_1_< 0.40) or moderate (*ICC*_1_= 0.40–0.60) reliability, but the symmetry ratio showed similar or better results.

The most reliable parameters were step time, single support time, double support time, walking speed, step length, and foot angle, all with *ICC*_10_ values greater than 0.80. The remaining parameters presented moderate (*ICC*_10_ = 0.40–0.60) or poor (*ICC*_10_ < 0.40) reliability. Conversely, when reliability was calculated for a single trial (*ICC*_1_), it was particularly poor, with only single support (>0.445), step length (>0.527), and foot angle (>0.477) showing moderate reliability, regardless of the symmetry calculation formula used. Therefore, it is strongly recommended not to rely on the calculation from a single trial when evaluating gait symmetry in Parkinson’s disease patients.

Regarding agreement, the Bland–Altman plots demonstrated consistent agreement among all pairs of symmetry indices as the difference between them largely fell within the confidence limits across all spatiotemporal parameters. In fact, for some symmetry equation pairs, the bias and Bland–Altman plots were similar or even identical.

The symmetry ratio (SR) equation is recommended for estimating gait symmetry in Parkinson’s disease patients. Among the five equations tested on this specific population, the SR was the most reliable and least variable index. Furthermore, the SR is simple, easy to calculate, and capable of distinguishing the limb with the most significant deviation. This recommendation aligns with the study conducted by Patterson, Gage, Brooks, Black, & McIlroy [11], who also recommend the symmetry ratio for calculating gait symmetry in stroke survivors. Their study found no significant differences among the four equations they examined and recommended using the SR due to its simplicity and clinical utility.

Nevertheless, Hill & Nantel [21] recently conducted a study exploring the sensitivity of symmetry metrics and concluded that ratio metrics, such as the SR, demonstrated low sensitivity as a measure of research power. It should be noted that their study only assessed the swing time parameter during gait on a split-belt treadmill in young adults, and simulation techniques were used to assess statistical power while mechanically influencing symmetry using a split-belt treadmill. Therefore, further research is necessary to explore the sensitivity of symmetry measurements using real data and under real-life conditions. These studies are crucial in drawing accurate conclusions about the sensitivity of symmetry indices when applied in clinical practice, particularly for evaluating neurological diseases.

One significant limitation of the examined symmetry equations is their potential to artificially inflate or deflate the level of asymmetry, which depends on the type of the specific parameter being studied or the magnitude of the asymmetry itself [2,14]. Additionally, it should be noted that the total of the referred symmetry indexes is not bounded. Furthermore, specific disadvantages include the different values produced using the symmetry ratio and ratio index equations depending on whether the greater value between the two lower limbs is used as the numerator or denominator, as well as the necessity for normalization to a reference value in the case of the symmetry index.

## 5. Conclusions

Based on the findings of the current study, the symmetry ratio exhibited lower variability and higher reliability compared with the other four indices across all spatiotemporal gait parameters. Therefore, the SR is recommended for estimating gait symmetry in Parkinson’s disease patients.

Lastly, except for step width and foot angle, all examined spatiotemporal parameters can yield consistent and reliable values for gait symmetry in individuals with Parkinson’s disease, provided that at least ten gait trials are recorded.

## Figures and Tables

**Table 1:** Indices for gait symmetry with underlying equations.

Indices Ratios	Equation
Symmetry Index	$SI=\frac{\left|X_{L}-\left.X_{R}\right|\right.}{0.5\;(\left|\left.X_{L}\right|\right.+\left|\left.X_{R}\right|\right.)}\times 100$
Gait Asymmetry	$GA=\left|\left.l_{n}\left(\frac{X_{L}}{X_{R}}\right)\right|\right.\times 100$
Symmetry Ratio	$SR=\frac{X_{L}}{X_{R}}$
Ratio Index	$RI=\left.\left|1-\frac{X_{L}}{X_{R}}\right.\right|\times 100$
Symmetry Angle	$SA=\left|\left.\frac{45^{{}^{\circ}}-arctan\left(\frac{X_{L}}{X_{R}}\right)}{90^{{}^{\circ}}}\right|\right.\times 100$

*X_L_* represents the values for the left limb and *Χ_R_* the value of the right limb for every parameter assessed.

**Table 2:** Mean and standard deviation (SD) symmetry values for all symmetry indices (SI, GA, SA, SR, RI) and spatiotemporal parameters.

	Cadence	Step Time	Stride Time	Single Support	Double Support	Walking Speed	Step Length	Stride Length	Step Width	Foot Angle
RI	1.74 (0.46)	6.25 (2.56)	1.72 (0.45)	6.24 (4.33)	7.27 (3.03)	2.2 (1.12)	5.81 (4.61)	2.14 (0.81)	7.94 (3.23)	92.69 (147.09)
SI	1.73 (0.45)	6.47 (2.82)	1.73 (0.45)	3.29 (16.9)	7.63 (3.39)	2.18 (1.08)	5.64 (4.07)	2.12 (0.77)	8.02 (3.13)	54.96 (39.13)
GA	1.73 (0.45)	6.47 (2.83)	1.73 (0.45)	5.97 (3.88)	7.64 (3.41)	2.18 (1.08)	5.65 (4.09)	2.12 (0.78)	8.03 (3.15)	65.57 (57.46)
SA	0.55 (0.14)	2.06 (0.9)	0.55 (0.14)	1.9 (1.23)	2.42 (1.08)	0.69 (0.34)	1.79 (1.29)	0.68 (0.25)	2.55 (0.99)	16.14 (10.52)
SR	1.01 (0.01)	0.96 (0.04)	0.99 (0.01)	1.04 (0.05)	0.95 (0.04)	1.01 (0.02)	1.01 (0.07)	1 (0.02)	0.99 (0.06)	1.54 (1.63)

**Table 3:** Bland–Altman mean values and 95% limits of agreement (lower, upper) for cadence, step time, stride time, single support, double support, and walking speed for each of the equation pairs (lower values indicate higher agreement). The components of each equation pair are displayed in the rows and columns, while the parameters are split on the left and right sides of the table.

		SI		SR		SA		GA		RI		
Cadence	SI			−0.019		−0.0012		−0.0010		−0.022		Step Time
				(−0.027, −0.010)		(−0.0019, −0.0004)		(−0.0004, −0.0024)		(−0.034, −0.011)		
	SR	−0.0045				0.018		0.020		−0.003		
		(−0.0063, −0.0027)				(0.010, 0.026)		(0.010, 0.030)		(−0.014, 0.007)		
	SA	0.00		0.0045				0.0022		−0.021		
		(0.00, 0.00)		(0.0027, 0.0063)				(0.0003, 0.0040)		(−0.032, −0.010)		
	GA	0.0002		0.0047		0.0002				−0.023		
		(0.0006, 0.0009)		(0.0031, 0.0062)		(0.0006, 0.0009)				(−0.035, −0.011)		
	RI	−0.011		−0.006		−0.011		−0.011				
		(−0.020, −0.002)		(−0.014, 0.002)		(−0.020, −0.002)		(−0.020, −0.002)				
Stride Time	SI			−0.008		0.0005		0.0009		0.019		Single Support
				(−0.032, 0.015)		(−0.0018, 0.0008)		(0.0004, 0.0015)		(0.012, 0.026)		
	SR	−0.0045				0.008		0.009		0.027		
		(−0.0063, −0.0027)				(−0.015, 0.031)		(−0.014, 0.033)		(0.003, 0.052)		
	SA	0.00		0.0045				0.0014		0.020		
		(0.00, 0.00)		(0.0027, 0.0063)				(0.0001, 0.0027)		(0.013, 0.027)		
	GA	0.0002		0.0047		0.0002				0.018		
		(−0.0006, 0.0009)		(0.0031, 0.0062)		(−0.0006, 0.0009)				(0.011, 0.025)		
	RI	0.011		0.015		0.011		0.011				
		(0.003, 0.019)		(0.006, 0.024)		(0.003, 0.019)		(0.003, 0.019)				
Double Support	SI			−0.007		−0.0003		0.0001		0.009		Walking Speed
				(−0.010, −0.003)		(−0.0011, 0.0006)		(−0.0005, 0.0006)		(0.002, 0.015)		
	SR	−0.021				0.0065		0.0068		0.015		
		(−0.029, −0.013)				(0.0033, 0.0097)		(0.0037, 0.0099)		(0.006, 0.025)		
	SA	−0.0014		0.020				0.0003		0.009		
		(−0.0027, −0.0001)		(0.013, 0.026)				(−0.0006, 0.0013)		(0.002, 0.015)		
	GA	0.0013		0.022		0.0028				0.009		
		(0.0004, 0.0023)		(0.014, 0.031)		(0.0011, 0.0044)				(0.002, 0.015)		
	RI	−0.023		−0.002		−0.022		−0.025				
		(−0.031, −0.015)		(−0.008, 0.004)		(−0.029, −0.014)		(−0.033, −0.016)				

**Table 4:** Bland–Altman mean values and 95% limits of agreement (lower, upper) for step length, stride length, step width, and foot angle for each of the equation pairs (lower values indicate higher agreement). The components of each equation pair are displayed in the rows and columns, while the parameters are split on the left and right sides of the table.

		SI		SR		SA		GA		RI		
Step Length	SI			−0.0065		−0.0002		0.0002		0.010		Stride Length
				(−0.0097, −0.0033)		(−0.0009, −0.0006)		(−0.0006, 0.0009)		(0.004, 0.016)		
	SR	−0.017				0.0063		0.10		0.016		
		(−0.026, −0.008)				(0.0032, 0.0095)		(−0.10, 0.31)		(0.007, 0.025)		
	SA	−0.0010		0.016				0.0003		0.010		
		(−0.0024, 0.0004)		(0.008, 0.024)				(−0.0006, 0.0013)		(0.004, 0.016)		
	GA	0.0011		0.018		0.0021				0.010		
		(0.0005, 0.0016)		(0.009, 0.027)		(0.0003, 0.0038)				(0.003, 0.016)		
	RI	0.023		0.040		0.024		0.022				
		(0.005, 0.042)		(0.014, 0.066)		(0.005, 0.043)		(0.004, 0.040)				
Step Width	SI			−0.11		−0.047		0.16		0.16		Foot Angle
				(−0.16, −0.06)		(−0.078, −0.016)		(−0.03, 0.036)		(−0.08, 0.41)		
	SR	−0.020				0.060		0.27		0.27		
		(−0.025, −0.014)				(0.035, 0.086)		(0.04, 00.50)		(0.00, 00.54)		
	SA	−0.0016		0.018				0.21		0.21		
		(−0.0026, −0.0006)		(0.013, 0.023)				(−0.02, 0.44)		(−0.05, 0.47)		
	GA	0.0013		0.021		0.0028				0.00		
		(0.0004, 0.0021)		(0.015, 0.027)		(0.0014, 0.0042)				(−0.25, 0.25)		
	RI	0.04		0.06		0.04		0.04				
		(0.00, 0.08)		(0.02, 0.10)		(0.01, 0.08)		(0.00, 0.08)				

## Data Availability

Not applicable.

## Supplementary Materials

The following supporting information can be downloaded at: https://www.mdpi.com/article/10.3390/neurolint15030070/s1, Figure S1. Bland–Altman plots showing the relationship between mean and difference, mean difference (continuous line) and the approximate 95% confidence intervals (dashed line), for each pair of symmetry indices (SI-SR, SI-SA, SI-GA, SI-RI, SR-SA, SR-GA, SR-RI, SA-GA, SA-RI, and GA-RI) for cadence, step time, and stride time. Figure S2. Bland–Altman plots showing the relationship between mean and difference, mean difference (continuous line) and the approximate 95% confidence intervals (dashed line), for each pair of symmetry indices (SI-SR, SI-SA, SI-GA, SI-RI, SR-SA, SR-GA, SR-RI, SA-GA, SA-RI, and GA-RI) for single support, double support, and walking speed. Figure S3. Bland–Altman plots showing the relationship between mean and difference, mean difference (continuous line) and the approximate 95% confidence intervals (dashed line), for each pair of symmetry indices (SI-SR, SI-SA, SI-GA, SI-RI, SR-SA, SR-GA, SR-RI, SA-GA, SA-RI, and GA-RI), for step length, stride length, step width, and foot angle.
