# Supplementary material for: A Comparative Analysis of Symmetry Indices for Spatiotemporal Gait Features in Early Parkinson’s Disease
Source: Neurol Int. 2023 Sep 7;15(3):1129–39. doi: 10.3390/neurolint15030070 (PMC10535875; doi:10.3390/neurolint15030070)
Supplement: Supplementary file 1 [file neurolint-15-00070-s001.zip › neurolint-2541167-supplementary.pdf]

Supplementary Material: A Comparative Analysis of Symmetry Indices for Spatiotemporal Gait Features in Early Parkinson's Disease

Erasmia Giannakou [1], Styliani Fotiadou [1,2], Vassilios Gourgoulis [1], Georgios Mavrommatis [3] and Nikolaos Aggelousis[1,*]

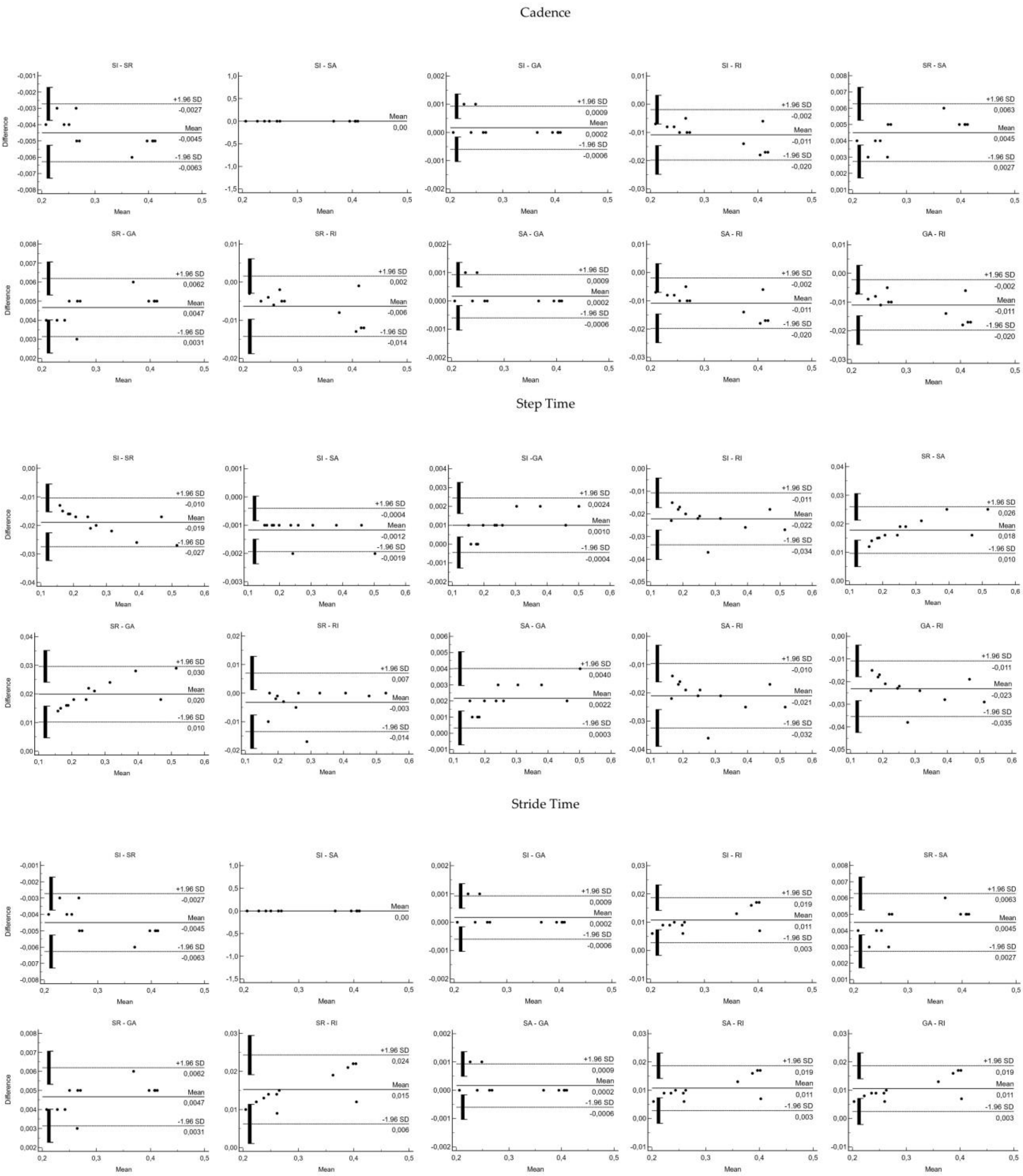

Figure S1. Bland-Altman plots of the relationship between mean and difference, mean difference (continuous line) and the approximate 95% confidence intervals (dashed line), of each symmetry

indices pair (SI-SR, SI-SA, SI-GA, SI-RI, SR-SA, SR-GA, SR-RI, SA-GA, SA-RI and GA-RI) for cadence, step time and stride time.

Single Support

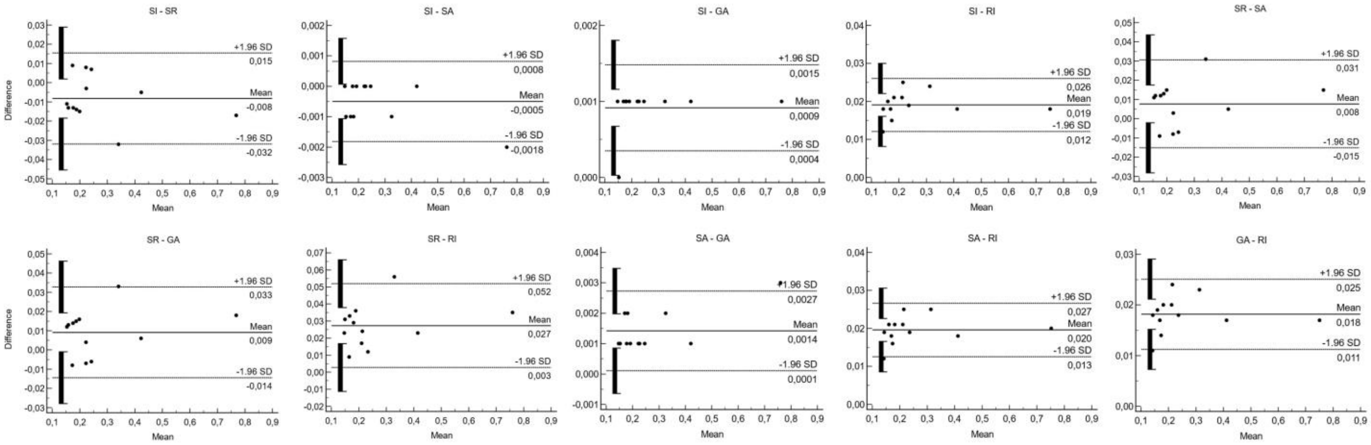

Double Support

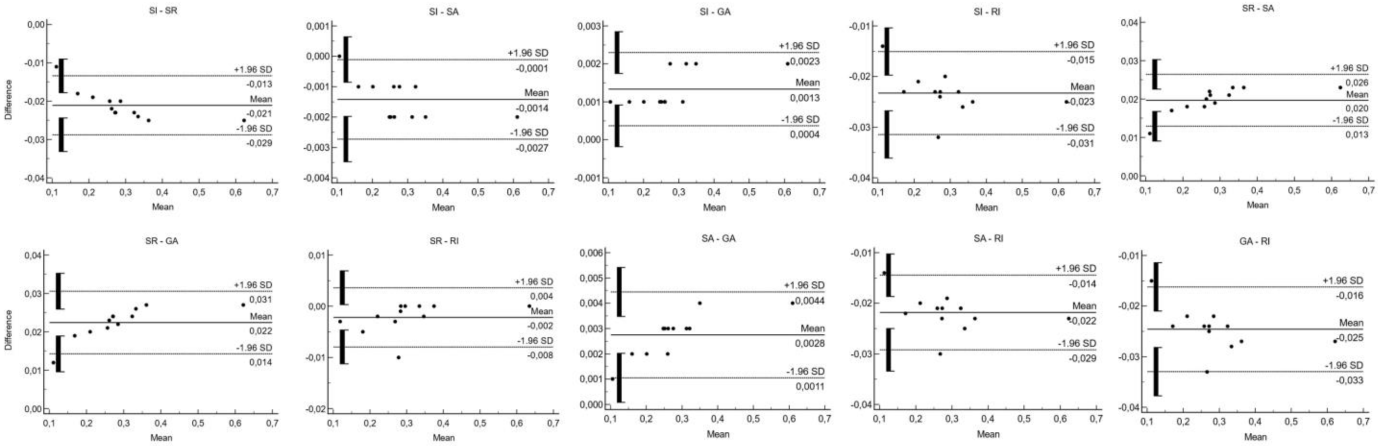

Walking Speed

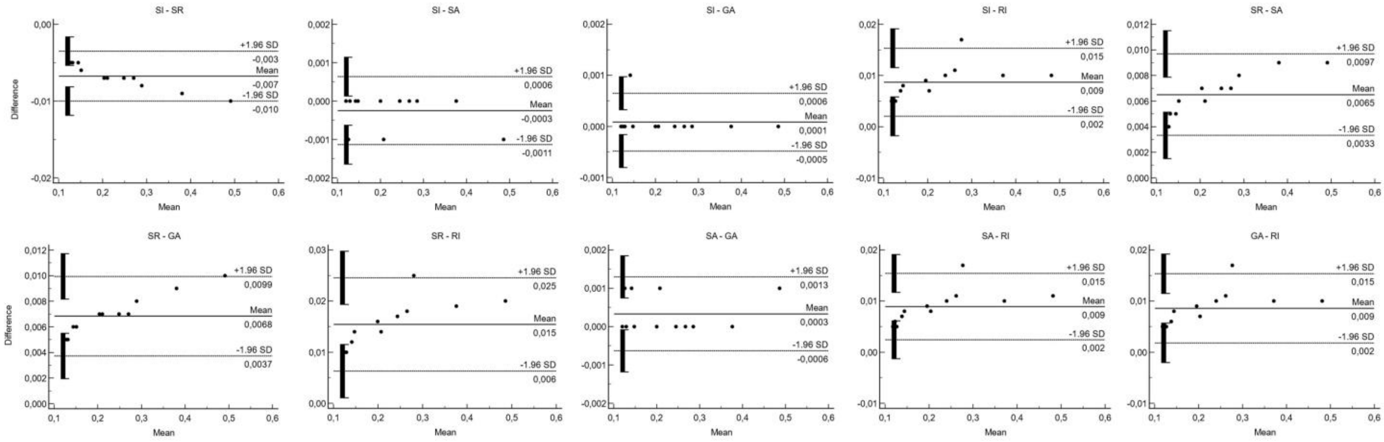

Figure S2. Bland-Altman plots of the relationship between mean and difference, mean difference (continuous line) and the approximate 95% confidence intervals (dashed line), of each symmetry indices pair (SI-SR, SI-SA, SI-GA, SI-RI, SR-SA, SR-GA, SR-RI, SA-GA, SA-RI and GA-RI) for single support, double support and walking speed.

Step Length

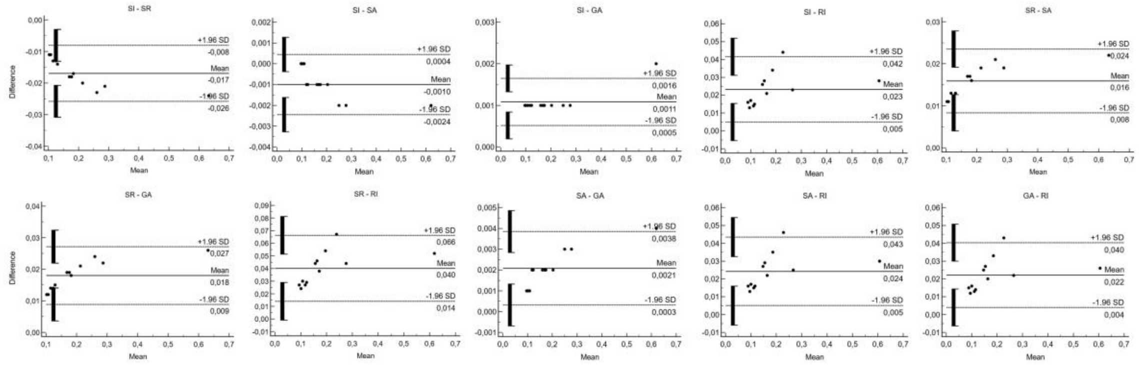

Stride Length

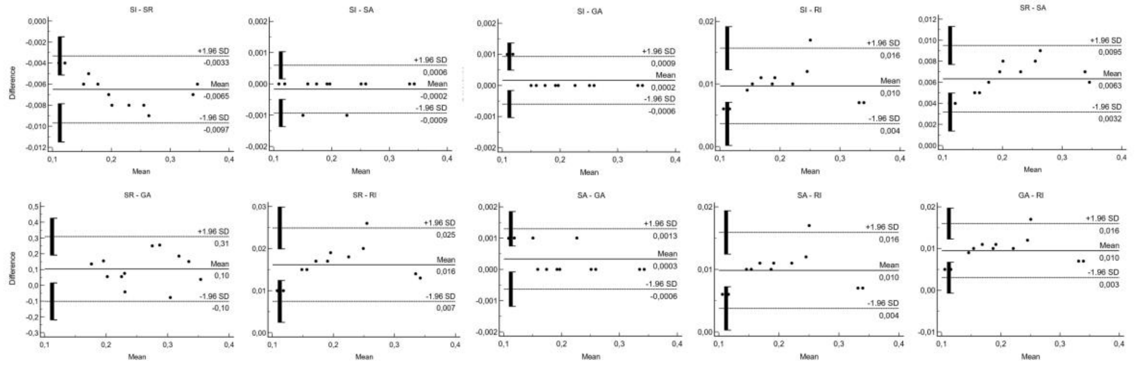

Step Width

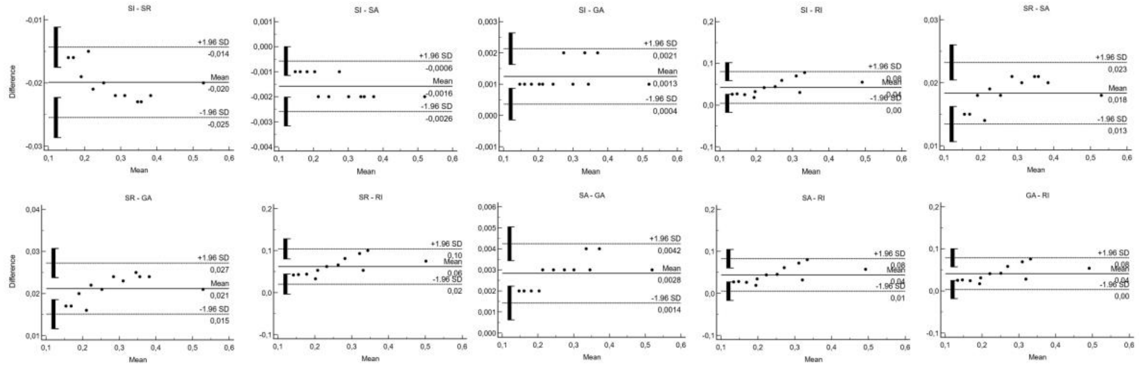

Foot Angle

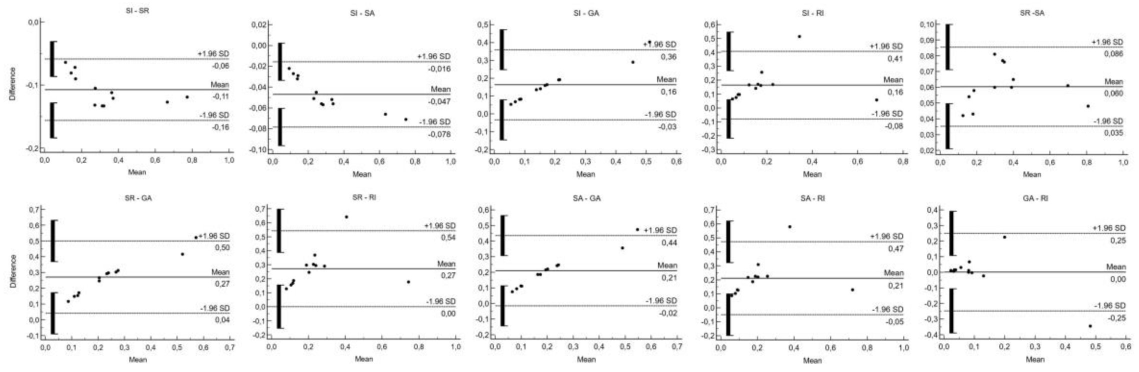

Figure S3. Bland-Altman plots of the relationship between mean and difference, mean difference (continuous line) and the approximate 95% confidence intervals (dashed line), of each symmetry indices pair (SI-SR, SI-SA, SI-GA, SI-RI, SR-SA, SR-GA, SR-RI, SA-GA, SA-RI and GA-RI) for step length, stride length, step width and foot angle.
